# Clinical characteristics and outcomes of patients with antibody‐related autoimmune encephalitis presenting with disorders of consciousness: A prospective cohort study

**DOI:** 10.1002/iid3.70019

**Published:** 2024-09-24

**Authors:** Dawei Shan, Huimin Zhang, Lili Cui, Shuting Chai, Weibi Chen, Gang Liu, Fei Tian, Linlin Fan, Le Yang, Yan Zhang

**Affiliations:** ^1^ Department of Neurology Xuanwu Hospital, Capital Medical University Beijing China

**Keywords:** anti‐N‐methyl‐D‐aspartate receptor encephalitis, autoimmune encephalitis, disorders of consciousness, outcome, risk factor

## Abstract

**Objective:**

To explore the clinical characteristics, short‐ and long‐term functional outcomes, and risk factors for antibody‐related autoimmune encephalitis (AE) in patients with disorders of consciousness (DoC).

**Methods:**

Clinical data were collected from AE patients admitted to Xuanwu Hospital of Capital Medical University from January 2012 to December 2021, and patients were followed up for up to 24 months after immunotherapy.

**Results:**

A total of 312 patients with AE were included: 197 (63.1%) with anti‐NMDAR encephalitis, 71 (22.8%) with anti‐LGI1 encephalitis, 20 (6.4%) with anti‐GABAbR encephalitis, 10 (3.2%) with anti‐CASPR2 encephalitis, 10 (3.2%) with anti‐GAD65 encephalitis, and 4 (1.3%) with anti‐AMPAR2 encephalitis. Among these patients, 32.4% (101/312) presented with DoC, and the median (interquartile range, IQR) time to DoC was 16 (7.5, 32) days. DoC patients had higher rates of various clinical features of AE (*p* < .05). DoC was associated with elevated lumbar puncture cerebrospinal fluid (CSF) pressure, CSF leukocyte count, and specific antibody titer (*p* < .05). A high percentage of patients in the DoC group had a poor prognosis at discharge and at 6 months after immunotherapy (*p* < .001), but no significant difference in prognosis was noted between the DoC group and the non‐DoC group at 12 and 24 months after immunotherapy. Dyskinesia (OR = 3.266, 95% CI: 1.550–6.925, *p* = .002), autonomic dysfunction (OR = 5.871, 95% CI: 2.574–14.096, and *p* < .001), increased CSF pressure (OR = 1.007, 95% CI: 1.001–1.014, *p* = .046), and modified Rankin scale (mRS) score ≥3 at the initiation of immunotherapy (OR = 7.457, 95% CI: 3.225–18.839, *p* < .001) were independent risk factors for DoC in AE patients.

**Conclusion:**

DoC is a relatively common clinical symptom in patients with AE, especially critically ill patients. Despite requiring longer hospitalization, DoC mostly improves with treatment of the primary disease and has a good long‐term prognosis after aggressive life support and combination immunotherapy.

## Graphical abstract

Our research explores the clinical characteristics and outcomes of autoimmune encephalitis (AE) in patients presenting with disorders of consciousness (DoC). We conducted a detailed analysis of 312 AE patients from Xuanwu Hospital, tracking various subtypes such as anti‐NMDAR and anti‐LGI1 encephalitis, and assessed their response to immunotherapy over a 24‐month period. The study highlights that while DoC significantly impacts early outcomes and requires intensive treatment, the long‐term prognosis for these patients can be favorable with appropriate and timely intervention.

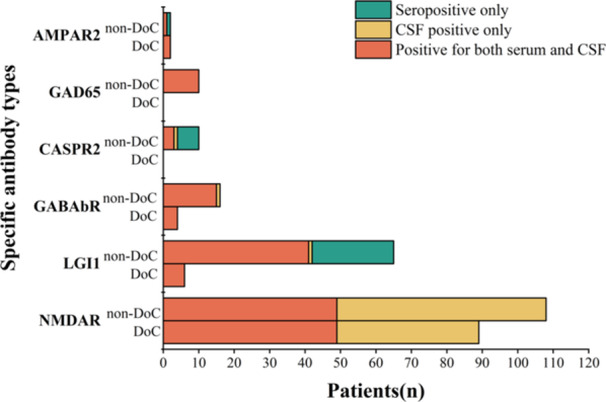

## 1 INTRODUCTION

Autoimmune encephalitis (AE) targets the neuronal surface or intracellular antigens and occurs at a rate of approximately 8–15 cases per 1,000,000 people annually.^1^ Among these, anti‐N‐methyl‐D‐aspartate receptor (anti‐NMDAR) encephalitis is the most prevalent form.^1^, ^2^ The inflammatory and immune response mechanisms underlying AE are pivotal in the pathogenesis and progression of the disease.^1^ Circulating neuronal cell surface antibodies target extracellular NMDAR proteins and have a direct pathogenic effect by affecting their function or causing their internalization without disrupting the cells.^3^, ^4^ Anti‐leucine‐rich glioma‐inactivated protein 1 (anti‐LGI1) encephalitis usually presents with mild faciobrachial dystonic or focal seizures followed by memory impairment.^5^ Anti‐contactin‐associated protein 2 (anti‐CASPR2) encephalitis is characterized by a long course of the disease.^6^ Anti‐ɑ‐amino‐3‐hydroxy‐5‐methyl‐4‐isoxazolepropionic acid receptor (anti‐AMPAR) encephalitis appears to be a recurrent limbic encephalitis.^7^, ^8^

First‐line drug therapy in the acute phase of AE includes corticosteroids and intravenous immunoglobulin (IVIG).^9^ Empirical intravenous methylprednisolone is a common method for inducing initial immunosuppressive and anti‐inflammatory effects in patients with AE.^10^ However, corticosteroids may lead to worsening of psychiatric symptoms in patients with AE, thus preventing timely assessment of the response to therapy.^9^ Patients with common comorbidities (e.g., hypertension or diabetes) may also have difficulty using corticosteroids.^9^ IVIG is a timely option for rapid immunomodulation.^11^ IVIG is associated with an increased risk of thromboembolism and may also exacerbate hyponatremia due to increased blood volume, which may lead to cerebral edema and worsened mental status.^9^, ^12^ If there is no significant clinical or imaging response to first‐line therapy after 2–4 weeks, second‐line agents with rapid and sustained immunosuppressive effects, including rituximab and cyclophosphamide, should be added.^11^ Rituximab is less toxic than cyclophosphamide and is therefore preferred. The side effects of rituximab are infusion‐related reactions and pulmonary infections.^13^ Cyclophosphamide can cause bone marrow suppression and infection.^9^ For refractory and relapsed patients, third‐line drug therapy, such as tocilizumab, has recently emerged but increases the risk of infection and hinders the recognition of infection.^14^ Studies in multiple drug trials are currently underway to confirm its efficacy and safety.^8^

Despite the favorable prognosis associated with early diagnosis and prompt immunotherapy, more than half of the patients with AE require intensive care unit (ICU) admission^11^, ^15^, ^16^ and present with critical clinical manifestations, such as disorders of consciousness (DoC), respiratory failure, status epilepticus (SE), and severe autonomic dysfunction. Moreover, the AE mortality rate is approximately 12%–40%.^17^, ^18^

Approximately 84% of patients with AE admitted to the ICU have DoC,^18^ including somnolence, sopor, coma, and confusion, which may be due to comorbid seizures,^19^ inflammation,^20^ increased intracranial pressure (ICP),^21^ and drug‐derived DoC. Patients with AE presenting with DoC are usually critically ill and require complex treatment, resulting in high human and financial costs and unknown long‐term prognoses; these conditions also affect treatment decisions and pose great ethical challenges.

Among patients with AE, some do not have a better prognosis than others for reasons that are unclear but that may be related to immune triggers and underlying pathogenic mechanisms.^22^ Despite the critical importance of DoC in the clinical course of AE, comprehensive studies systematically evaluating the prevalence, clinical characteristics, and long‐term outcomes of DoC in this patient population are scarce. The literature has focused primarily on the general clinical features and outcomes of AE, with limited attention given to the specific subset of patients with DoC.^23^, ^24^, ^25^ This highlights the need for focused research to clarify the distinct clinical characteristics and prognostic factors associated with DoC in AE patients. Thus, the results of this study may confirm the pathogenesis of antibody‐induced damage to neuronal function and other concomitant immune mechanisms contributing to neuronal damage in AE patients presenting with DoC. This information is critical for identifying patients who may benefit from more intensive immunotherapy and for developing treatments that may lead to improved outcomes.

Therefore, the aim of this single‐center prospective cohort study is to address this gap by systematically investigating the clinical characteristics and long‐term prognosis of AE patients presenting with DoC. By providing detailed insights into this critical aspect of AE, we aimed to inform clinical practice, improve patient management strategies, and ultimately enhance outcomes for this vulnerable patient population.

## 2 METHODS

### 2.1 Patients

In this study, data from patients with antibody‐related AEs who were admitted to the Department of Neurology of Xuanwu Hospital of Capital Medical University from January 2012 to December 2021 were prospectively collected and analyzed. The criteria for inclusion were as follows: (1) ≥14 years of age; (2) fulfillment of all three specified diagnostic criteria for AE^10^: (i) a subacute onset characterized by rapid progression within less than 3 months, manifesting as deficits in working memory (short‐term memory loss), altered mental status, or psychiatric disturbances; (ii) presence of at least one of the following: new focal findings in the central nervous system (CNS), seizures unexplained by any pre‐existing seizure disorders, cerebrospinal fluid (CSF) pleocytosis (more than five white blood cells per mm^3^), or magnetic resonance imaging (MRI) indications of encephalitis; (iii) reasonable exclusion of other potential causes; (3) detection of specific AE antibodies in the serum or CSF; and (4) consent obtained from the family of the patient. The exclusion criteria were as follows: no CSF antibody test via lumbar puncture was performed, and no immunotherapy was administered.

Patients fulfilling the inclusion criteria were categorized into two groups: those with DoC (DoC group) and those without DoC (non‐DoC group). The criteria specific to the DoC group included the following: (1) had a Glasgow coma score (GCS) <12, (2) had a DoC lasting >1 day and no episodic loss of consciousness, (3) had a DoC occurring during the current AE after excluding other etiologies as the cause, and (4) the effect of sedative medications was excluded from the determination of the time of onset of DoC.

### 2.2 Data collection

The prospectively collected data included patient demographic information (sex, age), clinical characteristics (type of AE‐specific antibodies, presence and specific manifestations of prodromal symptoms, clinical manifestations throughout the disease course and the presence or absence of concomitant tumors), ancillary findings (lumbar puncture CSF pressure, CSF white blood cell counts, CSF proteins, electroencephalogram (EEG), and cranial MRI findings), treatment information (admission to the ICU or not, respiratory‐assisted ventilation or not, time and drugs for initiating immunotherapy, therapeutic medications during the course of the disease, and number of hospitalization days), and prognostic evaluation. Specific antibodies against AE were detected in CSF and serum via indirect immunofluorescence test (IIFT) kits (EUROIMMUN AG), and antibody titers were recorded in detail and categorized as negative, weakly positive (1:10), positive (1:32, 1:100), or strongly positive (1:320). For patients in the DoC group, the duration from disease onset to the presentation of DoC, DoC duration, duration of mechanical ventilation, and degree of DoC were recorded. The degree of DoC was assessed by clinical physical examination, and the DoC state with the longest duration and the most severe impairment in consciousness during the patient's illness was selected to represent the degree of DoC in that patient. We chose the patient's GCS score at the time of the most severe impairment of consciousness as the DoC severity indicator.

### 2.3 Evaluation of treatment and prognosis

Every patient underwent tumor screening and received both immunotherapy and symptomatic care. The immunotherapy regimen included first‐line treatments such as glucocorticoid therapy, IVIG therapy (administered over 5 days at 0.4 g/kg), and/or plasma exchange. When needed, second‐line treatments involved the administration of immunosuppressants such as cyclophosphamide, rituximab, or mycophenolate mofetil.

The prognostic assessment of functional outcome was performed via the modified Rankin scale (mRS), with scores ranging from 0 (asymptomatic) to 6 (death), and the prognostic evaluation was based on the criteria of an mRS score <3 (good prognosis) versus an mRS score ≥3 (poor prognosis). All patients were assessed at the time of initiation of immunotherapy and upon discharge from the hospital and were followed up via telephone after discharge by a neurologist who was not informed about the study's design and the patient's condition, with follow‐ups at 6, 12, and 24 months after immunotherapy.

### 2.4 Data analysis and processing

The statistical analysis was performed via SPSS 26.0 (IBM Corporation) and R software (version 4.4.0). Images were plotted via OriginPro 2021 (OriginLab Corporation) and R software (version 4.4.0). Categorical variables are presented as counts (percentages). We assessed the distribution of continuous variables via the Kolmogorov‒Smirnov test and presented nonnormally distributed data as medians [interquartile ranges (IQRs)]. To compare categorical variables between groups, we employed either the Pearson *χ* ^2^ test or Fisher's exact test. The Mann‒Whitney *U* test was used for comparisons of nonnormally distributed variables between groups. All the statistical analyses were conducted via two‐tailed tests, with a *p* < .05 considered to indicate statistical significance. Variables that demonstrated a significant difference (*p* < .05) between the DoC and non‐DoC groups were selected and then screened according to clinical significance. To address multicollinearity and prevent overfitting in the high‐dimensional data set, LASSO regression was applied to the initial set of 21 variables within the cohort. Using threefold cross‐validation, the number of variables was reduced to 10 when log‐transformed lambda (log(λ)) reached a standard error (SE) within one SE of the minimum mean squared error for the log(λ). Then, the 10 variables were included and screened step‐by‐step in multifactorial logistic regression analysis via backward logistic regression to obtain independent risk factors for DoC. The calibration curve was depicted to demonstrate the fit between the predicted and observed probabilities. The predictive role of independent risk factors for DoC was evaluated via receiver operating characteristic (ROC) curves and the concordance index (C‐index). The utility of the model in a clinical setting was evaluated via decision curve analysis (DCA), which quantifies the net benefit at various threshold probabilities. The final model was subjected to 10‐fold cross‐validation and bootstrap validation.

## 3 RESULTS

### 3.1 Patient characteristics

**Figure 1:**
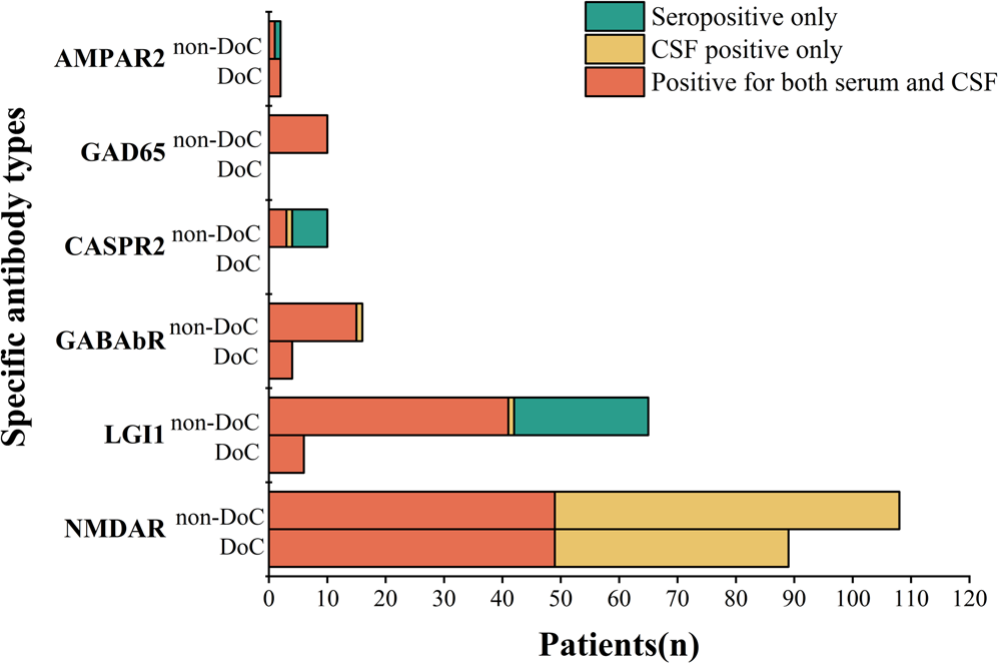
Results of different specific antibodies in the CSF and serum of enrolled patients. AMPAR, ɑ‐amino‐3‐hydroxy‐5‐methyl‐4‐isoxazolepropionic acid receptor; CASPR2, contactin‐associated protein 2; CSF, cerebrospinal fluid; DoC, disorders of consciousness; GABAbR, gamma‐aminobutyric acid receptor type b; GAD65, glutamate decarboxylase 65; LGI 1, leucine‐rich glioma inactivated 1; NMDAR, N‐methyl‐D‐aspartate receptor.

A total of 312 patients diagnosed with antibody‐mediated AEs were included in the study. In total, 170 (54.5%) patients were male, and the median (IQR) age at onset was 34.5 (24, 57) years. As shown in Table 1, patients in the DoC group had a lower median age at disease onset than did those in the non‐DoC group (26 vs. 39 years, *p*< .001). The results for different specific antibodies detected in the CSF and serum of the enrolled patients are shown in Figure 1.

**Table 1:** Demographic data, clinical symptoms, and auxiliary test results for patients with antibody‐associated autoimmune encephalitis.

	Total (*n* = 312)	DoC group (*n* = 101)	Non‐DoC group (*n* = 211)	*p* Value
Sex, *n* (%)				.621
Male	170 (54.5)	53 (52.5)	117 (55.5)	
Female	142 (45.5)	48 (47.5)	94 (44.5)	
Age, years, median (IQR)	34.5 (24, 57)	26 (21, 39.5)	39 (27, 61)	＜.001
AE specific antibody types, *n* (%)				
NMDAR	197 (63.1)	89 (88.1)	108 (51.2)	＜.001
LGI1	71 (22.8)	6 (5.9)	65 (30.8)	＜.001
GABAbR	20 (6.4)	4 (4)	16 (7.6)	.222
CASPR2	10 (3.2)	0 (0)	10 (4.7)	.060
GAD65	10 (3.2)	0 (0)	10 (4.7)	.060
AMPAR2	4 (1.3)	2 (2)	2 (0.9)	.597
Presence of prodromal symptoms, *n* (%)	125 (40.1)	57 (56.4)	68 (32.2)	＜.001
Fever	66 (21.2)	32 (31.7)	34 (16.1)	.002
Headache	29 (9.3)	12 (11.9)	17 (8.1)	.276
Respiratory symptoms	22 (7.1)	9 (8.9)	13 (6.2)	.375
Emesis	3 (1)	2 (2)	1 (0.5)	.246
Diarrhea	2 (0.6)	2 (2)	0 (0)	.104
Dizziness	3 (1)	0 (0)	3 (1.4)	.554
Clinical manifestations, *n* (%)				
Language impairment	72 (23.1)	35 (34.7)	37 (17.5)	.001
Seizures	212 (67.9)	79 (78.2)	133 (63)	.007
Status epilepticus	14 (4.5)	13 (12.9)	1 (0.5)	＜.001
Mental behavior disorders	198 (63.5)	91 (90.1)	107 (50.7)	＜.001
Movement disorders	111 (35.6)	70 (69.3)	41 (19.4)	＜.001
Autonomic dysfunction	71 (22.8)	54 (53.5)	17 (8.1)	＜.001
Cognitive impairment	186 (59.6)	70 (69.3)	116 (55)	.016
Tumor comorbidity, *n* (%)	24 (7.7)	12 (11.9)	12 (5.7)	.055
Lumbar puncture CSF analysis				
Opening pressure, mmH_2_O, median (IQR)	160 (130, 203.8)	190 (150, 235)	150 (125, 190)	＜.001
CSF WBC, ×10^6^/L, median (IQR)	8 (2, 23)	19 (6, 37)	5 (1, 15)	＜.001
CSF protein, mg/dL, median (IQR)	34.4 (24, 45.7)	28 (19, 43)	35 (27, 46)	.001
CSF specific antibody titers, *n* (%)				
Negative	30 (9.6)	0 (0)	30 (14.2)	＜.001
Weakly positive (1:10)	49 (15.7)	5 (5)	44 (20.9)	＜.001
Positive (1:32, 1:100)	207 (66.3)	76 (75.2)	131 (62.1)	.021
Strongly positive (1:320)	26 (8.3)	20 (19.8)	6 (2.8)	＜.001
Serum specific antibody titers, *n* (%)				
Negative	102 (32.7)	40 (39.6)	62 (29.4)	.072
Weakly positive (1:10)	52 (16.7)	25 (24.8)	27 (12.8)	.008
Positive (1:32, 1:100)	153 (49)	32 (31.7)	121 (57.3)	＜.001
Strongly positive (1:320)	5 (1.6)	4 (4)	1 (0.5)	.039
Electroencephalogram abnormalities *n* = 300, *n* (%)	215 (71.7)	88 (88)	127 (63.5)	＜.001
Cranial MRI abnormalities, *n* (%)	204 (65.4)	53 (52.5)	151 (71.6)	.001

Abbreviations: AE, autoimmune encephalitis; AMPAR, ɑ‐amino‐3‐hydroxy‐5‐methyl‐4‐isoxazolepropionic acid receptor; CASPR2, contactin‐associated protein 2; CSF, cerebrospinal fluid; DoC, disorders of consciousness; GABAbR, gamma‐aminobutyric acid receptor type b; GAD65, glutamate decarboxylase 65; IQR, interquartile range; LGI 1, leucine‐rich glioma inactivated 1; MRI, magnetic resonance imaging; NMDAR, N‐methyl‐D‐aspartate receptor; WBC, white blood cell.

**Figure 2:**
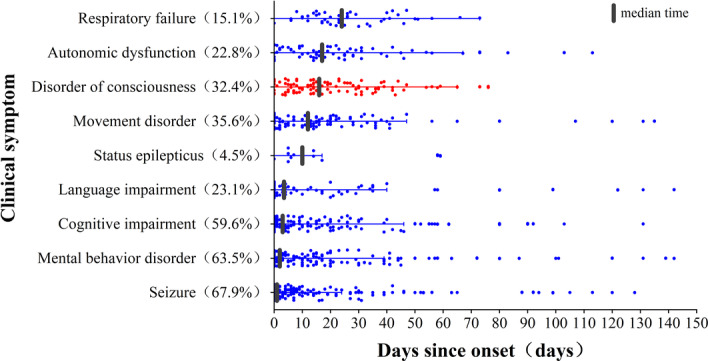
Temporal progression of clinical symptoms. Median time and interquartile range from onset to the first appearance of each clinical symptom in the enrolled AE patients. The percentages show the frequency of each clinical symptom in the cohort of AE patients.

The time between onset and the appearance of each clinical symptom, ranked according to the median (IQR) time, in the patients in this study was as follows: seizure [1 (0, 10) day], mental behavior disorder [2 (0, 16.25) days], cognitive impairment [3 (0, 19.25) days], language impairment [3.5 (0, 22.5) days], SE [10 (5, 14.75) days], movement disorders [12 (0, 22) days], DoC [16 (7.5, 32) days], autonomic dysfunction [17 (6, 31) days], and respiratory failure [24 (16, 40) days] (Figure 2). Among the different antibody‐mediated AEs, a significant variation was observed in the proportion of patients with anti‐NMDAR encephalitis between the two groups on the basis of the occurrence of DoC (88.1% vs. 51.2%, *p* < .001), and these patients were more likely to develop DoC.

### 3.2 DoC patient characteristics

The overall incidence of DoC in this study was 32.4% (101/312). As shown in Table 2, patients with DoC varied in their degree of impaired consciousness, with the largest percentage of patients experiencing light coma (37.6%). The median (IQR) GCS score was 8 (5, 11), with the highest percentage of patients (48.5%) having a score of 9–11. A total of 5.9% (6/101) of patients had DoC at disease onset. The median (IQR) duration from onset to the presentation of DoC and the median (IQR) time span of DoC were 16 (7.5, 32) and 31 (20, 62) days, respectively, and the duration of DoC exceeded 30 days in 52.5% (53/101) of patients. A total of 46.5% (47/101) of DoC patients were treated with mechanical ventilation, and the median (IQR) duration for mechanical ventilation was recorded at 35 (22, 70) days. Sedative medication was used in 43.6% (44/101) of patients with DoC, and 58.5% (31/53) of patients with DoC lasting >30 days were sedated.

**Table 2:** Characteristics of 101 antibody‐related AE patients with DoC.

	DoC group (*n* = 101)
Days from onset to DoC, days, median (IQR)	16 (7.5, 32)
DoC duration days	
Days, median (IQR)	31 (20, 62)
Days, minimum/maximum	3/158
≤7 days, *n* (%)	10 (9.9)
8–30 days, *n* (%)	38 (37.6)
＞30 days, *n* (%)	53 (52.5)
Mechanical ventilation duration days	
Days, median (IQR)	35 (22, 70)
Days, minimum/maximum	2/252
GCS score	
Median (IQR)	8 (5, 11)
Minimum/maximum	3/11
3–5, *n* (%)	26 (25.7)
6–8, *n* (%)	26 (25.7)
9–11, *n* (%)	49 (48.5)
Degree of DoC, *n* (%)	
Somnolence	14 (13.9)
Sopor	6 (5.9)
Confusion	30 (29.7)
Light coma	38 (37.6)
Moderate coma	11 (10.9)
Deep coma	2 (2)
Use of sedative drugs, *n* (%)	44 (43.6)
Proportion of patients with DoC lasting ≤7 days who are on sedatives, *n* = 10	3 (30)
Proportion of patients with DoC lasting 8–30 days who are on sedatives, *n* = 38	10 (26.3)
Proportion of patients with DoC lasting ＞30 days who are on sedatives, *n* = 53	31 (58.5)

Abbreviations: DoC, disorders of consciousness; GCS, Glasgow Coma Scale; IQR, interquartile range.

### 3.3 Comparison of clinical and ancillary characteristics between patients with and without DoC

As shown in Table 1, patients with DoC had greater incidences of febrile prodromal symptoms, respiratory failure, speech disorders, seizures, SE, psychiatric symptoms, movement disorders, autonomic dysfunction, and cognitive deficits than patients without DoC (*p* < .05). DoC was associated with elevated lumbar puncture CSF pressure, CSF leukocyte count, and specific antibody titer (*p* < .05). A greater percentage of DoC patients presented with EEG abnormalities.

### 3.4 Comparison of therapy and prognosis between patients with and without DoC

**Figure 3:**
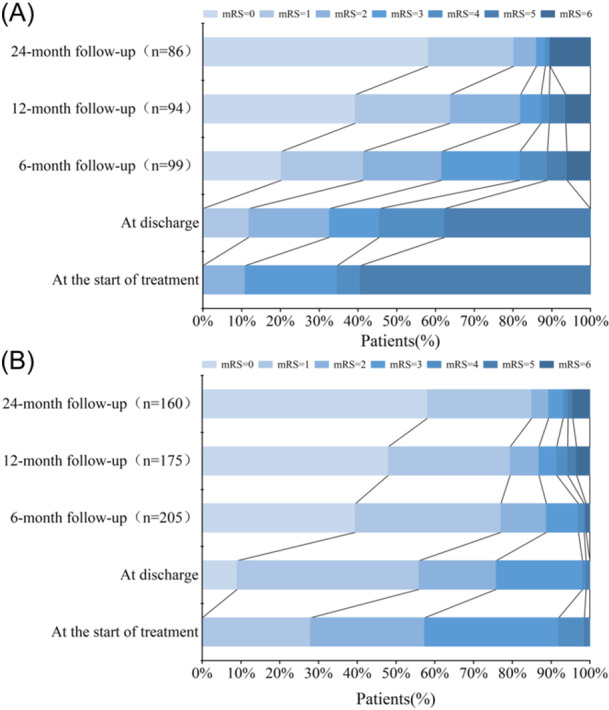
Comparison of mRS scores at the start of immunotherapy, at discharge, and at 6, 12, and 24 months after immunotherapy in the DoC group (A) versus the non‐DoC group (B). The number of patients followed‐up during each period is in parentheses on the vertical axis, and the decrease indicates the number of patients lost to follow‐up. mRS, modified Rankin scale.

As shown in Table 3, compared with non‐DoC patients, a significantly greater percentage of DoC patients required ICU admission and ventilator support (75.2% vs. 0.9%, *p*< .001; 46.5% vs. 0%, *p* < .001), and the median (IQR) duration of hospitalization was longer [31 (18.5, 57) days versus 14 (10, 18) days, *p* < .001]. In addition, patients with DoC experienced a shorter duration from onset to the initiation of immunotherapy; a greater proportion of DoC patients were administered glucocorticoid‐IVIG combination therapy; and a greater proportion of DoC patients received glucocorticoid, IVIG, plasma exchange, or immunosuppressant therapy during the course of the disease (*p *< .05). A significant difference in prognosis was noted between the two groups at discharge and at 6 months after immunotherapy, with a high percentage of patients having a poor prognosis in the DoC group (67.3% vs. 24.2%, *p*< .001; 38.4% vs. 11.2%, *p* < .001); however, no significant difference in prognosis was noted between the two groups at 12 and 24 months after immunotherapy (Figure 3).

**Table 3:** Treatment and outcomes for patients with antibody‐related autoimmune encephalitis.

	Total (*n* = 312)	DoC group (*n* = 101)	Non‐DoC group (*n* = 211)	*p* Value
ICU admission, *n* (%)	78 (25)	76 (75.2)	2 (0.9)	＜.001
Ventilator‐assisted breathing, *n* (%)	47 (15.1)	47 (46.5)	0 (0)	＜.001
Initial immunotherapy, *n* (%)				
Steroids	225 (72.1)	61 (60.4)	164 (77.7)	.001
IVIG	72 (23.1)	30 (29.7)	42 (19.9)	.055
Steroids + IVIG	15 (4.8)	10 (9.9)	5 (2.4)	.009
Days from onset to start of immunotherapy, days, median (IQR)	30 (15, 60.8)	20 (10, 33)	40 (20, 92)	＜.001
Steroids, *n* (%)	281 (90.1)	96 (95)	185 (87.7)	.042
IVIG, *n* (%)	166 (53.2)	86 (85.1)	80 (37.9)	＜.001
Plasma exchange, *n* (%)	48 (15.4)	46 (45.5)	2 (0.9)	＜.001
Immunosuppressants, *n* (%)	84 (26.9)	43 (42.6)	41 (19.4)	＜.001
Days of hospitalization, days, median (IQR)	16 (12, 24)	31 (18.5, 57)	14 (10, 18)	＜.001
mRS at the initiation of immunotherapy, *n* (%)				＜.001
＜3	132 (42.3)	11 (10.9)	121 (57.3)	
≥3	180 (57.7)	90 (89.1)	90 (42.7)	
mRS at discharge, *n* (%)				＜.001
＜3	193 (61.9)	33 (32.7)	160 (75.8)	
≥3	119 (38.1)	68 (67.3)	51 (24.2)	
mRS at 6 months after immunotherapy, *n* = 304, *n* (%)				＜.001
＜3	243 (79.9)	61 (61.6)	182 (88.8)	
≥3	61 (20.1)	38 (38.4)	23 (11.2)	
mRS at 12 months after immunotherapy, *n* = 269, *n* (%)				.277
＜3	229 (85.1)	77 (81.9)	152 (86.9)	
≥3	40 (14.9)	17 (18.1)	23 (13.1)	
mRS at 24 months after immunotherapy, *n* = 246, *n* (%)				.440
＜3	217 (88.2)	74 (86)	143 (89.4)	
≥3	29 (11.8)	12 (14)	17 (10.6)	

Abbreviations: DoC, disorders of consciousness; ICU, intensive care unit; IQR, interquartile range; IVIG, intravenous immunoglobulin; mRS, modified Rankin scale.

### 3.5 Potential risk factors associated with the development of DoC in patients with AE

**Figure 4:**
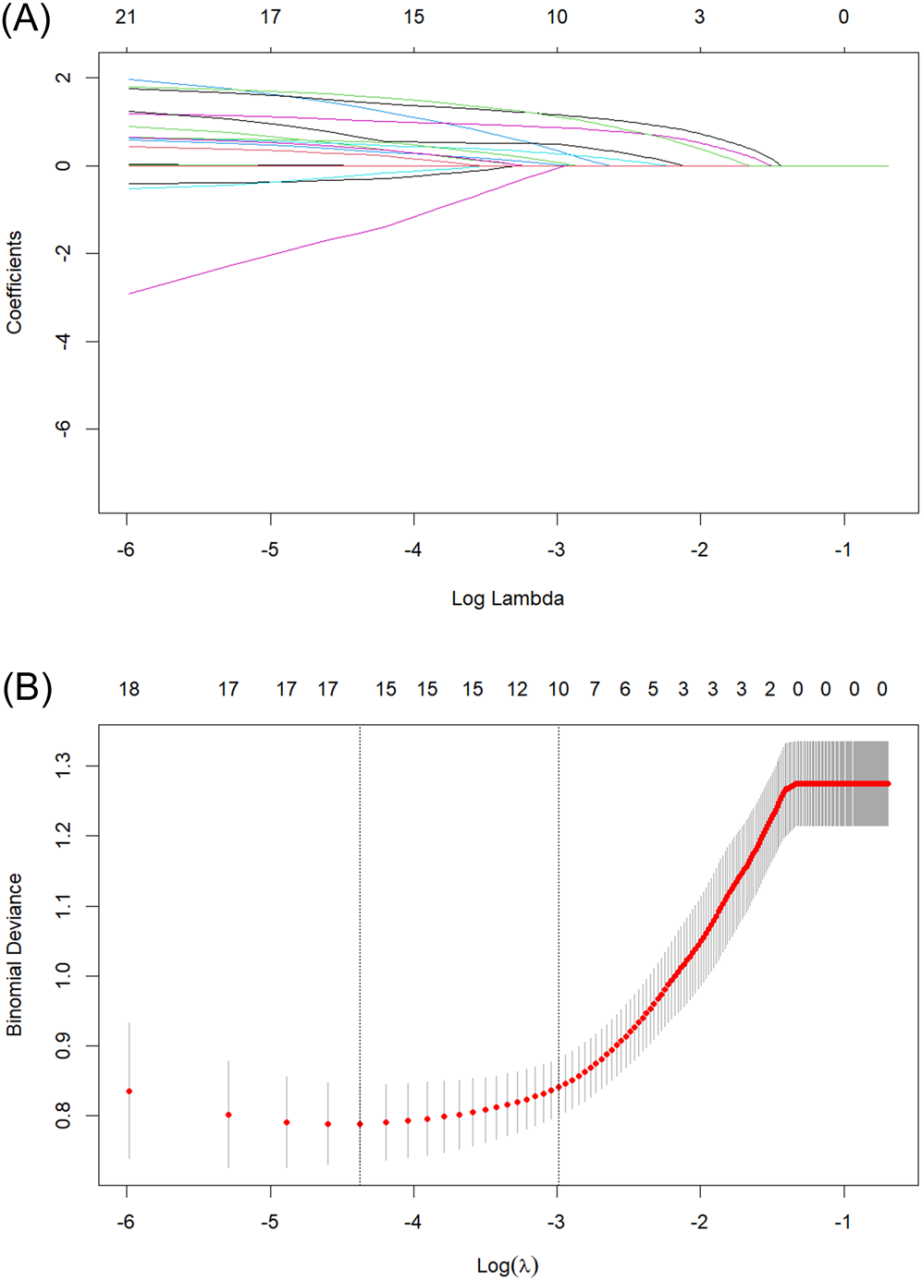
LASSO regression curves. (A) Curve of the regression coefficient versus log lambda; (B) curve of MSE versus log lambda. λmin represents the cutoff point at which the MSE takes the minimum value, whereas λ1se represents the point where the MSE takes a value of 1 × standard error. MSE, mean squared error.

LASSO regression was applied to the initial set of 21 variables within the cohort. Using threefold cross‐validation, the number of variables was reduced to 10: SE, psychiatric symptoms, dyskinesia, abnormalities in autonomic function, lumbar puncture pressure, negativity for CSF‐specific antibodies, strong positivity for CSF‐specific antibodies, EEG abnormalities, diagnosis of anti‐NMDAR encephalitis, and mRS score at the initiation of immunotherapy (Figure 4). Four independent risk factors were then derived via multifactorial logistic regression.

As shown in Table 4, the multifactorial logistic regression analysis revealed that dyskinesia (OR = 3.266, 95% CI: 1.550–6.925, *p* = .002), abnormalities in autonomic function (OR = 5.871, 95% CI: 2.574–14.096, *p* < .001), increased lumbar puncture pressure (OR = 1.007, 95% CI: 1.001–1.014, *p* = .046), and mRS score ≥3 at the initiation of immunotherapy (OR = 7.457, 95% CI: 3.225–18.839, *p* < .001) were independent risk factors for DoC in patients with AE.

**Table 4:** Multivariate logistic regression analyses of potential predictive factors for patients with DoC.

Variable	*p* Value	Odds ratios (95% CI)
Dyskinesia	.002	3.266 (1.550‐6.925)
Abnormalities of autonomic function	<.001	5.871 (2.574–14.096)
Increased lumbar puncture pressure	.046	1.007 (1.001–1.014)
mRS ≥3 at initiation of immunotherapy	<.001	7.457 (3.225–18.839)

Abbreviations: CI, confidence interval; DoC, disorders of consciousness; mRS, modified Rankin scale.

**Figure 5:**
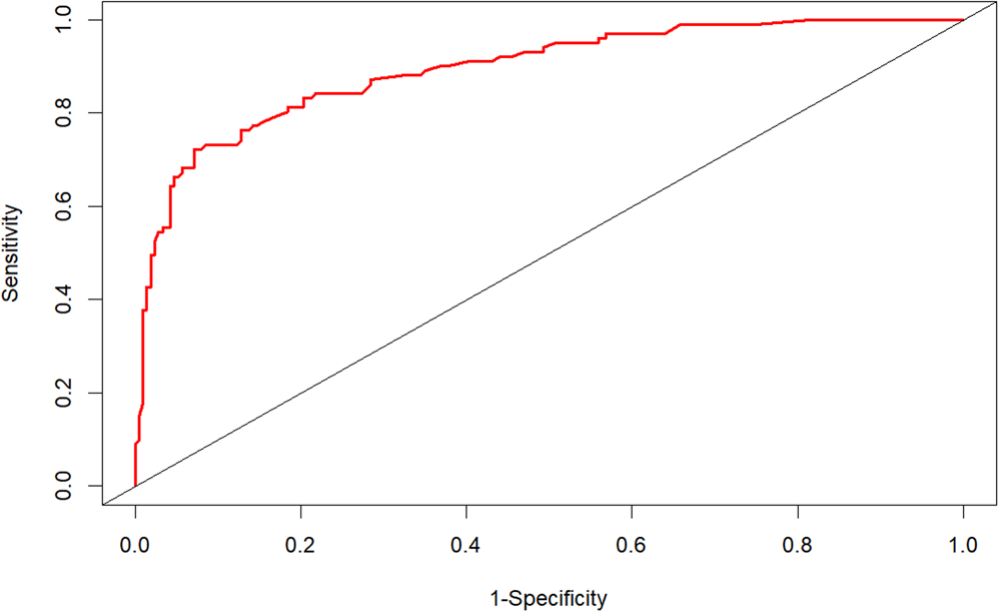
ROC curve. ROC curve for evaluating the role of the above four variables in predicting the occurrence of DoC in AE patients.

**Figure 6:**
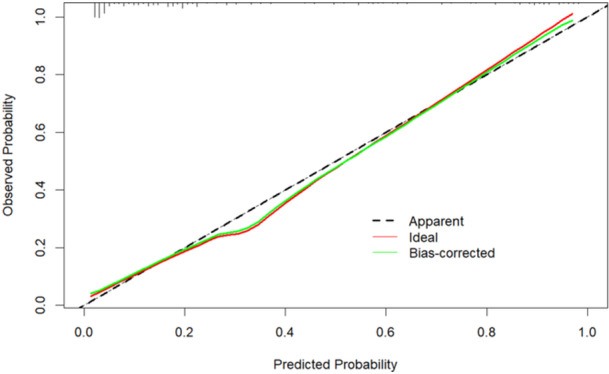
Calibration curves for DoC prediction. Curve of the observed probability versus the predicted probability.

**Figure 7:**
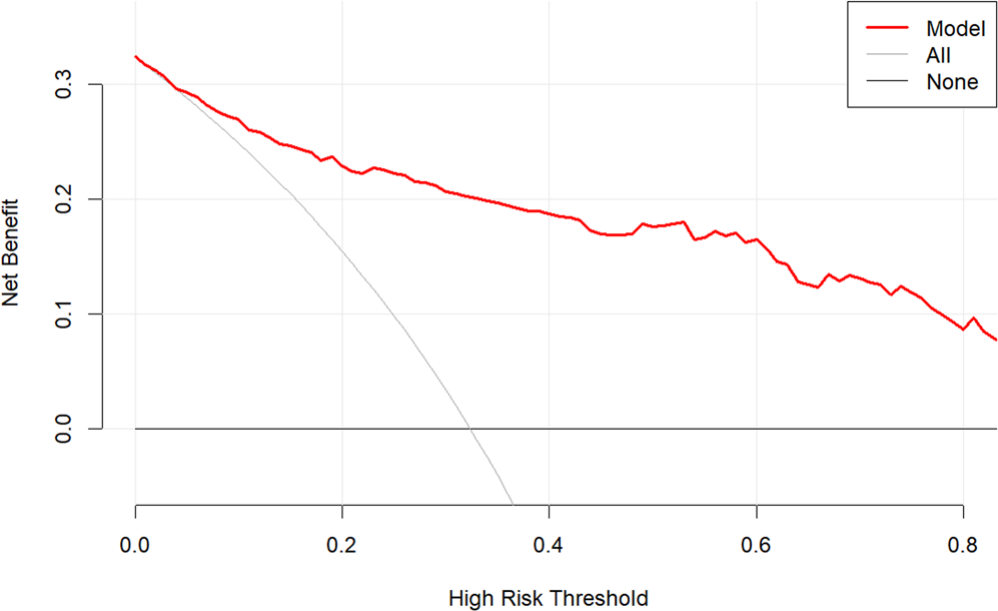
Decision curve analysis of DoC prediction. Curve of the net benefit versus high risk threshold. This curve reflects a high net benefit value of the model for predicting the presence of DoC in AE patients.

The ROC curve was used to assess the predictive capacity of the aforementioned four parameters for the DoC in patients with AE. The area under the curve (AUC) and the C‐index were 0.894 (95% CI: 0.855–0.932) (Figure 5). The calibration curve was illustrated to indicate a good fit between the predicted and observed probabilities (Figure 6). DCA demonstrated a significant net benefit of the predictive model in the cohort (Figure 7). The final model was subjected to 10‐fold cross‐validation and bootstrap validation. The mean AUC obtained from the 10‐fold cross‐validation was 0.884 (95% CI: 0.875–0.894), and that from the bootstrap validation was 0.891 (95% CI: 0.889–0.894). These findings indicate that the internal validation of the data set is consistently reproducible.

## 4 DISCUSSION

AE comprises a group of diseases that require early and definitive diagnosis and active treatment; a high percentage of patients are critically ill,^18^ and DoC is a relatively common clinical manifestation. The percentage of AE patients who presented with DoC in previous studies ranged from approximately 28.1%–57%,^26^, ^27^ and 32.4% of patients with AE exhibited DoC in the current study. The presence of DoC often represents a more severe condition and serves as an independent risk factor for disease severity in AE patients upon admission,^28^ requiring aggressive transfer to the ICU. This study revealed that the proportions of DoC patients who were admitted to the ICU, who were receiving mechanical ventilation and who had abnormal lumbar puncture pressure and CSF antibody titer results were greater than those in the non‐DoC group. These findings suggest that patients with DoC have more severe disease and should be given greater priority in the clinic.

In our study, we observed the time from onset to each clinical manifestation in all patients and reported that DoC was usually a late‐onset clinical feature with a median time of 16 days, with most DoC symptoms appearing after the development of epilepsy and psychotic symptoms and before autonomic abnormalities and respiratory failure, which aligns with the results of a prior study.^29^ The reason why DoC appears in the later stages is unclear. Irani et al.^29^ hypothesized that symptoms such as seizures and psychiatric abnormalities in the early stages are due to dysfunction of cortical neurons. It is the secondary synthesis of specific antibodies in the later stages of the sheaths and the formation of oligoclonal strips that may be responsible for wider cerebral involvement, such as when there is subcortical dysfunction and white matter lesions. The fact that DoC occurs mostly in late stages may be due to damage to the brain region (discussed below) by secondary synthesis of specific antibodies in the sheaths in the later stages of the disease. Several studies^30^, ^31^ have also demonstrated this through the temporal relationship between longitudinal changes in EEG and disease progression. These findings provide us with great clinical inspiration to pay close attention to emerging clinical features in AE patients and to the disease stage of the patient. For example, when a patient develops dyskinesia, it is important to be alert to the possibility of subsequent DoC and to administer the necessary and timely life‐supporting treatment to help the patient during this dangerous period.

The causes and specific mechanisms of DoC in patients with AE have not been determined. It is hypothesized^19^, ^20^, ^21^ that this may be due to damage to the brainstem and thalamus from the primary disease combined with SE, increased ICP, persistent hyperthermia due to infection, or the effects of sedative medications. Complex interactions among multiple neurotransmitter systems in the brainstem, hypothalamus, thalamus, and basal forebrain play crucial roles in regulating cortical arousal.^32^ DoC is characterized by disruptions in the neural pathways that maintain arousal and consciousness.^33^ The brain network connectivity hypothesis states that when neuronal activity related to consciousness is severely disrupted, functional connectivity in the default mode network responsible for internal consciousness regulation is reduced.^33^, ^34^, ^35^ Antibody‐mediated inflammation due to primary disease may induce neuronal deficits and synaptic plasticity dysfunction^20^; when inflammation involves consciousness‐related neural pathways, such as the thalamus and the reticular activating system, there may be disruptions in neuronal and brain network connections, which subsequently lead to DoC. Another hypothesis emphasizes the role of the nucleus reticularis thalami (nRT), a key structure in the dorsal thalamus that maintains arousal.^36^ In patients with anti‐NMADR encephalitis, overall NMDAR‐dependent nRT hypofunction leads to a predominance of δ rhythms throughout the cerebral cortex, resulting in decreased levels of consciousness.^32^, ^36^, ^37^ In one study,^38^ resting‐state functional magnetic resonance imaging (rs‐fMRI) was used to measure intrinsic neural activity in the brains of AE patients who presented with DoC syndrome. The results revealed a disruption in the functional connectivity of the patients' brain networks, and fMRI was synchronized with improvements in consciousness, demonstrating that antibody‐mediated disruption of neuronal signaling in the NMDAR led to disruption of intrinsic neural activity in the patients' brains, which was associated with severely impaired consciousness. An EEG‐related study also noted that the more severe the degree of EEG abnormalities in AE patients was, the greater the rate of DoC.^31^ A high degree of EEG abnormality may indicate that many abnormal functional areas of the brain are involved and that many consciousness‐related neural pathways are involved, leading to a high rate of DoC. In this study, a greater proportion of EEG abnormalities was observed in patients in the DoC group than in those in the non‐DoC group, and the classification of EEG severity and multistage EEG monitoring should be performed in future studies to further explore the correlation between DoC and EEG findings in patients with AE.

Seizures may occur in patients with various types of AEs, and seizures and SEs are usually acute symptomatic manifestations of inflammatory processes in the brain.^39^ The DoC in some patients is due to the presence of SE or nonconvulsive SE (NCSE), and EEGs can help to definitively determine these causes in these patients.^19^ In this study, the percentage of patients who experienced SE in the DoC group was significantly greater than that in the non‐DoC group, which supports the above reasoning. However, the overall number of patients with SE was low, which may be biased, and the sample size should be further increased in future studies. Prompt administration of medication to control SE after it develops has been identified as an important measure, and prompt control of the primary cause of encephalitis through immunomodulation will contribute to a rapid recovery of consciousness.

Inflammatory reactions in the brains of AE patients and SEs can trigger cerebral edema, which is followed by an increase in the ICP, and severe ICP elevation can lead to DoC.^40^ In this study, the lumbar puncture pressure was significantly greater in the DoC group than in the non‐DoC group, suggesting that cerebral edema was more severe in patients with DoC and that an increased ICP secondary to cerebral edema may impact patient consciousness. Mammele et al.^21^ reported a case of rapidly progressing anti‐NMDAR encephalitis in the presence of DoC in a patient with abnormally early development of cerebral edema, swift progression to brain herniation, and brain death within 3 days from onset. Although this may be a rare complication of AE, clinicians should be aware of the possibility of an acute increase in the ICP in AE patients presenting with DoC. Patients with AE usually have abnormal immune function, which increases the risk of infection, and persistent hyperthermia after infection can lead to hyperventilation, hypocapnia, and even DoC.^28^, ^41^ In summary, the possible causes of DoC in patients with AE are diverse and should be carefully analyzed according to the patient's condition in the clinic and actively corrected so that the patient can regain consciousness as soon as possible.

Several previous studies of anti‐NMDAR encephalitis have shown that a certain percentage of patients develop DoC, but comparisons between different antibodies against AE have been limited.^20^, ^26^, ^27^, ^28^, ^31^, ^42^ Anti‐NMDAR encephalitis is the most common type of AE and accounts for approximately 54%–80% of AEs.^2^ In this study, we found that patients with anti‐NMDAR encephalitis had a greater likelihood of developing DoC. The potential reasons are as follows. First, anti‐NMDAR encephalitis is consistent with diffuse encephalitis, with a large area of the brain involved, and it has the most diverse symptoms.^43^ Clinical symptoms mirror the anatomical distribution of NMDARs, which are broadly expressed across the brain. The attachment of specific antibodies triggers the internalization of synaptic NMDARs, thereby diminishing NMDAR‐associated synaptic currents.^38^ DoC occurs when the neural pathways involved in the connectivity of the conscious brain network are disrupted. In contrast, patients with AE caused by other antibodies in this study usually had limbic encephalitis, where the affected brain regions were located mainly in the limbic system. The patients manifested with mental behavioral abnormalities, seizures, and short‐term memory deficits as the main deficits^10^, ^39^, ^44^ and with regions related to consciousness seldom being involved; therefore, DoC was less frequently observed. Second, a greater percentage of patients diagnosed with anti‐NMDAR encephalitis (approximately 42.8%) were adolescents.^45^ A study reported a negative correlation between age at onset and seizures in patients with anti‐NMDAR encephalitis^45^; thus, patients with anti‐NMDAR encephalitis are more likely to develop seizures. As mentioned earlier, SE may be one of the reasons why patients develop DoC; therefore, it is hypothesized that this is one of the reasons why patients with anti‐NMDAR encephalitis are more likely to develop DoC. Therefore, while managing patients with anti‐NMDAR encephalitis, more attention should be given to changes in consciousness than when managing patients with other types of AEs.

Sedative medications are commonly used during treatment, and this study revealed a correlation between prolonged DoC duration and the use of sedative medications. The use of sedative medications can impact the level of consciousness and may prolong the DoC course and length of hospitalization^46^; however, the use of sedative medications is necessary in some cases. A total of 91.3% of patients with anti‐NMDAR encephalitis benefitted from midazolam administered via various routes, and the rate of adverse effects was low.^47^ Several studies have shown the great value of sedative medications for a wide range of symptoms in patients with AE.^47^, ^48^, ^49^ Intravenous midazolam and phenobarbital are more effective at controlling SE and refractory SE in patients with AE.^47^ Midazolam can be the drug of choice for treating AE movement disorders.^48^ The efficacy of the combination of two sedative drugs for the control of paroxysmal sympathetic hyperactivity (PSH) is significant.^49^ Patients with AE often experience agitation and mental disorders when consciousness is restored, at which point sedative medications are often required^46^ and can prolong the DoC. If the patient's condition progresses, it would be counterproductive to forcibly discontinue sedative medications to regain consciousness; active symptomatic treatment to help the patient through the natural course of the disease is key.

Cranial MRI is an important test for visualizing the location and extent of lesions in patients with AE. Several studies have shown^18^, ^20^, ^50^ that the presence or absence of abnormalities on cranial MRI does not significantly correlate with the prognosis of patients with AE. However, there are few reports on whether variability in cranial MRI abnormalities occurs in patients with different severities of AE, and the conclusions are mixed.^28^, ^51^ In our research, we observed that the percentage of patients with DoC who presented with abnormalities on cranial MRI was low, which may be attributed to the following reasons. First, cranial MRI findings may vary depending on the AE disease stage.^45^ One study revealed that the proportion of abnormalities in patients with AE was elevated after a second cranial MRI,^29^ which may affect the accuracy of the analysis. Second, conventional 3.0T MRI scans are unable to reveal abnormal changes in the cerebral cortex and neural networks in the early stages of AE.^28^ Third, some patients with AE may have no abnormalities on routine MRI, and more than 50% of patients with anti‐NMDAR encephalitis have no significant abnormalities on routine MRI,^52^, ^53^ which potentially impacts the results. Most of the MRI examinations of the DoC patients in this study were completed before the occurrence of DoC, and secondary MRI examinations were not performed after the occurrence of DoC given the severity of the patient's medical conditions and the use of ventilators, possibly contributing to the low percentage of abnormalities found on cranial MRI. Even if patients are usually rechecked via cranial MRI after stabilization, the accuracy of the results may be affected by the low detection rate of routine MRI. This problem can be addressed in the future by increasing the sample size, analyzing cranial MRI data according to the stage of the disease, and evaluating the results via high‐field‐strength MRI or fMRI.

The prognosis for patients with AE accompanied by DoC is frequently a matter of concern for physicians and patients' families. Several previous studies on risk factors for AE prognosis^18^, ^20^, ^26^, ^27^, ^42^ have shown that DoC could be a risk factor for adverse outcomes in patients with AE at an earlier stage (≤12 months after onset). However, no studies have evaluated the difference in short‐ and long‐term prognoses associated with the presence of DoC in patients with AE. In this study, patients in both groups were followed up for up to 24 months, and patients in the DoC group had a worse short‐term prognosis (≤6 months after immunotherapy) than patients in the non‐DoC group did, whereas no significant difference in long‐term prognosis (≥12 months after immunotherapy) was observed. The poorer short‐term prognosis of DoC patients may be related to the poor response to early immunotherapy in critically ill patients, the slow improvement in their condition, and the various complications that occur during hospitalization. Approximately half of the DoC patients in this study were mechanically ventilated. Prolonged bed rest and mechanical ventilation may lead to numerous complications, including pulmonary infections, urinary tract infections, deep vein thrombosis, ICU‐acquired myasthenia gravis, and multiple organ dysfunction syndrome (MODS),^20^, ^54^, ^55^ which can affect the short‐term prognosis of DoC patients. Although some AE patients who presented with DoC still experienced poor short‐term prognoses after prompt immunotherapy, no significant difference in long‐term prognosis was observed, and the majority of patients still improved or recovered completely, which aligns with the results of prior research.^11^, ^56^ Thus, it is possible that we administered more rapid and aggressive immunotherapy to patients in the DoC group. Studies on the pathogenesis of anti‐NMDAR encephalitis have shown that, after active immunotherapy to eliminate autoantibodies against NMDARs, NMDARs are reactivated, thus restoring synaptic currents,^57^ and most patients' symptoms, such as psychiatric symptoms, dyskinesias, and DoC, improve, except for some residual cognitive deficits,^38^, ^57^ which may explain the above findings at the cellular level. These findings revealed that for AE patients with prolonged DoC, good life support and early administration of combination immunotherapy may still lead to a good long‐term prognosis, even if the condition is critical and the duration of the disease is very long.

Considering that this was a prospective cohort study conducted at a single center, several limitations should be noted. First, the majority of patients in the cohort were diagnosed with anti‐NMDAR encephalitis, potentially biasing the results. Second, DoC is a risk factor for prolonged cognitive impairment in ICU patients surviving severe infections,^58^ and altered functional connectivity of brain networks and white matter integrity during recovery from AE may contribute to the persistence of cognitive deficits.^38^ We assessed patient prognosis exclusively via the mRS score and did not perform a more detailed assessment via other cognitive function scales; therefore, the impact of the DoC on long‐term prognosis may have been underestimated. Third, blood neutrophils and lymphocytes and their ratio reflect the severity of AE,^28^ and cytokines and chemokines in CSF and serum are also involved in the pathogenesis of AE.^59^ Neutrophil, lymphocyte, cytokine, and chemokine levels were not assessed in this study to explore the correlation between the above indicators and DoC. Despite its limitations, this study serves as a valuable exploration of AE patients who present with DoC and could serve as a reference for future research; larger multicenter clinical studies are needed to confirm these conclusions.

## 5 CONCLUSIONS

In summary, DoC is a relatively common clinical feature in patients with AE, and such patients have more severe conditions and longer hospitalization durations. The presence of DoC is linked to a worse short‐term prognosis and requires immediate recognition, and these patients should be administered more aggressive immunotherapy. With life support and adherence to combination immunotherapy, DoC patients mostly improve after treatment for the primary disease and have a good long‐term prognosis.

## AUTHOR CONTRIBUTIONS

Dawei Shan collected, analyzed, and visualized the data and wrote the manuscript. Lili Cui, Weibi Chen, Gang Liu, Fei Tian, and Linlin Fan enrolled patients. Huimin Zhang, Shuting Chai, and Le Yang collected the clinical data. Yan Zhang conceived and designed the study, reviewed the data interpretation and supervised the manuscript. All the authors reviewed the final manuscript.

## CONFLICT OF INTEREST STATEMENT

The authors declare no conflict of interest.

## ETHICS STATEMENT

All patients or their representatives provided informed consent. The Ethics Committee of Xuanwu Hospital, Capital Medical University, approved this study (No. 2022‐104), which complied with the principles of the Declaration of Helsinki.

## Data Availability

Anonymized data of this study are available on reasonable request.
